# Reaction norm modeling of high‐dimensional genomic and environmental data improves prediction accuracy in winter wheat

**DOI:** 10.1002/tpg2.70332

**Published:** 2026-10-27

**Authors:** Shailesh Raj Acharya, Julian Garcia‐Abadillo, Jeanette Lyerly, Gina Brown‐Guedira, Diego Jarquin, Nonoy Bandillo

**Affiliations:** ^1^ Department of Crop and Soil Sciences North Carolina State University Raleigh North Carolina USA; ^2^ Department of Agronomy University of Florida Gainesville Florida USA; ^3^ USDA‐ARS SEA, Plant Science Research Raleigh North Carolina USA

## Abstract

Genomic prediction models that account for genotype‐by‐environment (G × E) have the potential to accelerate the rate of genetic gain for yield and agronomic performance, yet relatively few studies have applied G × E prediction in public soft red winter wheat (*Triticum aestivum*) breeding programs. In this study, we extended a reaction norm‐based genomic prediction framework by integrating weather‐based environmental covariates to more effectively capture G × E interactions. Key agronomic traits, including seed yield, plant height, test weight, and heading date, were evaluated across 25 environments (location–year) using over 3200 breeding lines from the North Carolina State University small grains breeding program. Multiple genomic prediction models were compared using several cross‐validation (CV) schemes representing common breeding scenarios. Across traits, the reaction norm M5 model, which incorporates both G × E and genotype‐by‐environmental covariate interactions (G × O), achieved the highest prediction accuracy (PA) in CV2 (predicting incomplete field trials) and CV1 for yield and test weight (predicting new lines). The highest PA was observed for test weight under CV2 (0.55) and for yield under CV1 (0.41). Under CV0 (predicting new environments), the M3 model incorporating G × E produced the highest PA across traits, with the greatest accuracy for plant height (0.45), although differences among M2, M3, and M4 were small. Prediction under CV00 (predicting new lines in new environments) remained more challenging, with PA values 0.10–0.20 across traits. Overall, our results demonstrate that integrating environmental covariates into genomic prediction models can improve predictive performance across diverse wheat‐growing environments in North Carolina, supporting their utility for applied breeding efforts.

## Core Ideas

- Integrating genotype‐by‐environment (G × E) interactions with environmental covariates improves prediction accuracy in winter wheat.
- Reaction norm models effectively capture G × E interactions, but prediction accuracy depends on the target breeding scenarios.
- Provides a practical framework for improving genomic prediction and selection across diverse environments.

## Plain Language Summary

This study explores how adding environmental information to genomic prediction models can improve prediction accuracy in a public winter wheat breeding program. Using about 3200 breeding lines evaluated in 25 environments across diverse conditions in North Carolina, we evaluated statistical models that capture how different wheat lines respond to changing environments. By incorporating weather data, we improved the ability to predict performance across locations and years. These findings provide practical insights for refining selection strategies and accelerating genetic gain in wheat breeding.

**Abbreviations**
BLUE: best linear unbiased estimate
CV: cross validation
G × E: genotype‐by‐environment
G × O: genotype‐by‐environmental covariate
GBLUP: genetic best linear unbiased prediction
GBS: genotype‐by‐sequencing
GS: genomic selection
IID: independently and identically distributed
MSE: mean squared error
PA: prediction accuracy
RNM: reaction norm model
SNP: single nucleotide polymorphism
SRWW: soft red winter wheat

## 1 INTRODUCTION

Soft red winter wheat (*Triticum aestivum* L.; SRWW) constitutes the largest class of wheat cultivated in the southeastern United States. The region produces approximately 9.31 million metric tons of SRWW annually, with North Carolina contributing ∼0.49 million metric tons, representing an estimated $100 million economic contribution to the state's agricultural economy (USDA, 2025). North Carolina wheat production supports diverse end uses, including livestock feed (∼80%), milling and baking (∼20%), and emerging specialty markets, providing economic value to farmers, seed companies, and other stakeholders. Beyond feed production, North Carolina–grown SRWW varieties are utilized for food products such as crackers, cookies, pastries, and other confectionery applications.

Wheat production in North Carolina spans across diverse environments characterized by substantial variation in temperature, precipitation, soil properties, and management practices. This environmental heterogeneity creates complex genotype‐by‐environment (G × E) interactions that challenge breeders’ ability to accurately evaluate genetic potential and develop cultivars with both high yield performance and broad adaptation. Therefore, understanding and effectively modeling G × E interactions is critical for improving selection accuracy and accelerating the development of stable, high‐performing SRWW cultivars.

Over the past two decades, genomic selection (GS) has emerged as a transformative tool in crop breeding (Crossa et al., 2010; Meuwissen et al., 2001). By leveraging genome‐wide molecular markers to predict genomic estimated breeding values, GS accelerates genetic gain by enabling earlier identification of superior lines and reducing the need for extensive multi‐environment field evaluations (Atanda & Bandillo, 2024; Bandillo et al., 2023; Saludares et al., 2024). This is particularly useful when evaluating complex traits such as grain yield, across multiple environments which is both costly and time intensive (Bandillo et al., 2023; Saludares et al., 2024). Despite these advantages, conventional genomic prediction models such as Genomic Best Linear Unbiased Prediction (GBLUP) focus on main additive genetic effects which assumes that genetic performance remains constant across environments, effectively treating G × E interactions as unexplained noise rather than important biological source of information (Burgueño et al., 2012; Jarquín et al., 2014). In public breeding programs targeting diverse production regions characterized by substantial year‐to‐year and location‐specific climatic variation, ignoring G × E can reduce prediction accuracy and limit the ability to identify wheat cultivars that are either broadly adapted or specifically suited to specific environments (Crossa et al., 2017).

To overcome these limitations, reaction norm model (RNM) was introduced as an extension of the GBLUP framework (Jarquín et al., 2014). Unlike traditional models that treat environment as a discrete uncorrelated category, RNM describes genotype performance as a continuous function across environmental gradients. This allows the models to better capture how different genotypes respond to changing environmental conditions. Jarquín et al. (2014) established the statistical mechanics of RNMs for GS using high‐dimensional genomic and environmental data in wheat. Because explicitly modeling G × E interactions between thousands of short nucleotide polymorphisms (SNPs) and numerous environmental covariates (EC) introduces extreme computational dimensionality, Jarquín et al. (2014) parameterized these interactions through variance–covariance structures using the Hadamard product (element‐wise multiplication) of genomic and environmental relationship matrices. This framework partitions the total genetic variance into two components—the main additive genetic effects and genotype‐specific reaction norm slopes, which describe how individual genotype differs in their sensitivities to environmental variations.

The RNM framework has proven to be highly flexible and has been successfully applied across diverse crops, traits, and environmental descriptors. In multi‐environmental wheat trials, Jarquín et al. (2017) showed that incorporating macro‐climatic weather variables into RNM significantly improved genomic prediction accuracy by 20%–30% compared to standard main‐effect models. Beyond weather data, Canella Vieira et al. (2022) showed that integrating soil‐derived covariates into RNM effectively captured micro‐environmental variability resulting in improved prediction of soybean grain yield across different breeding scenarios. Beyond agronomic traits, Brault et al. (2025) applied RNMs and joint regression models using ECs to predict Fusarium head blight resistance in spring wheat, showing that environmental sensitivity can also be effectively modeled for complex disease resistance traits. More recently, McBreen et al. (2025) demonstrated that integrating high throughput phenomics data into multi‐environmental RNMs improved the ability to capture environmental variation across southeastern US wheat breeding trials.

In this study, we leveraged a large multi‐environment dataset consisting of more than 3200 SRWW breeding lines evaluated across 25 environments from the North Carolina State University small grains breeding program. Using this extensive dataset, we implemented a reaction norm modeling framework to evaluate genomic prediction accuracy for four key agronomic traits, including grain yield, test weight, heading date, and plant height. We compared five prediction models across four cross‐validation schemes (CV2, CV1, CV0, and CV00) representing increasingly challenging breeding scenarios, from predicting new lines in known environments to predicting new lines in completely untested environments (Burgueño et al., 2012; Jarquín et al., 2014). Our objective was to determine whether explicitly modeling G × E interactions and incorporating weather‐based environmental covariates can improve prediction accuracy and enable more effective GS under diverse production environments.

## 2 MATERIALS AND METHODS

### 2.1 Plant materials and phenotypic data

Phenotypic data for four agronomic traits—grain yield, test weight, heading date, and plant height—were collected from 3200 SRWW breeding lines evaluated across eastern, central, and western North Carolina from 2017 to 2023, with an average replication of two per line. The breeding lines consisted of a diverse range of lines from observational, preliminary, and advanced yield trials.

Yield was observed across all 25 environments with the minimum number of eight genotypes in CLAYTON2018 and the maximum of 419 in KINSTON2021. The total number of environments and their respective number of genotypes across these environments for grain yield is shown in Figure 1.

**FIGURE 1:**
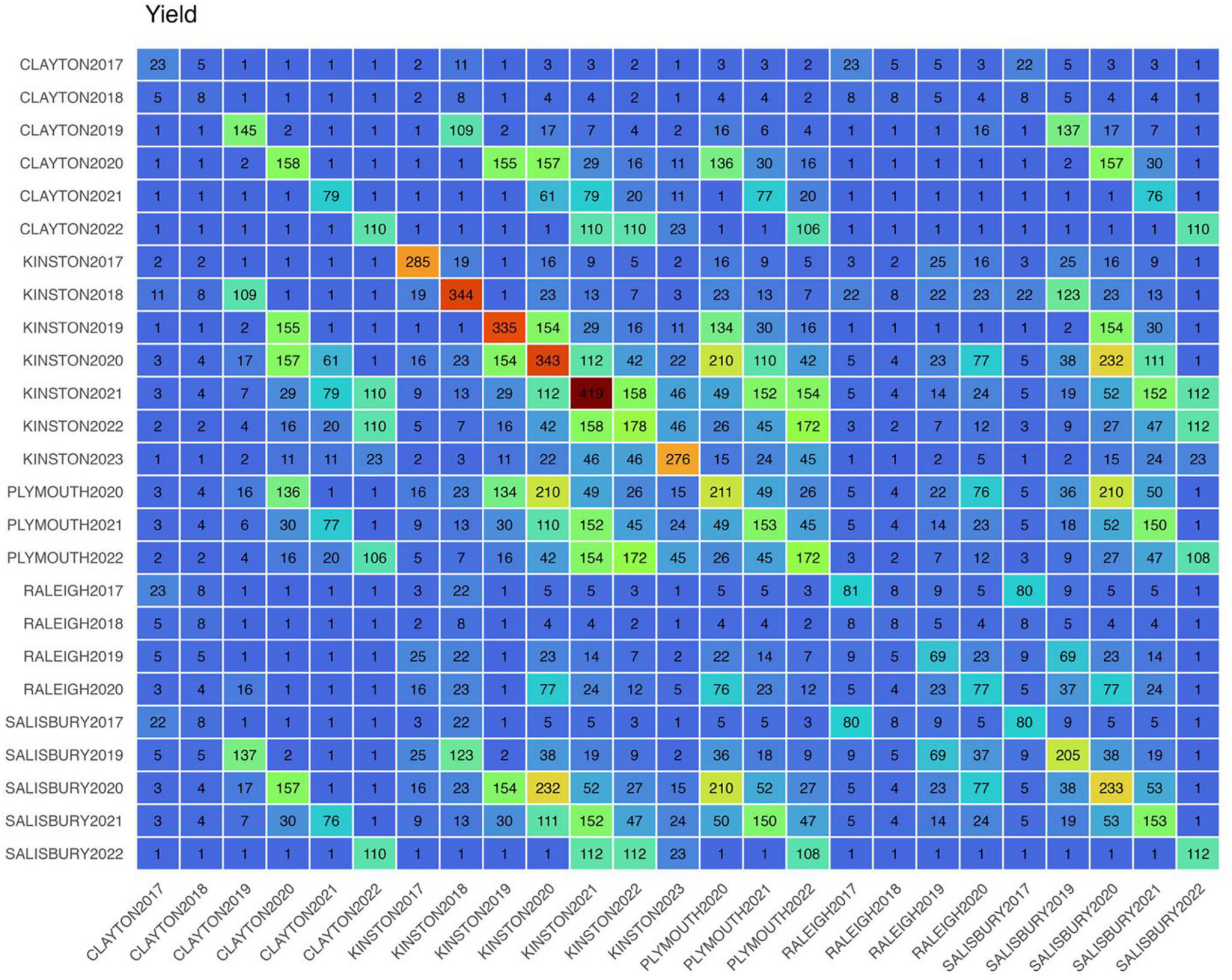
Total number of overlapping genotypes between environments for Yield. Yield was observed across all 25 environments. The 25 × 25 matrix shows the number of lines in each environment (location–year combinations) on the diagonal. The upper off‐diagonal shows common lines between environments, while the lower off‐diagonal shows lines not shared between environments.

Similarly, the total number of environments tested for all remaining traits—test weight, heading date, and plant height is presented in Table 1, and their environment‐specific genotype number in a similar matrix form is present in Figure S1.

**Core Ideas**

- Integrating genotype‐by‐environment (G × E) interactions with environmental covariates improves prediction accuracy in winter wheat.
- Reaction norm models effectively capture G × E interactions, but prediction accuracy depends on the target breeding scenarios.
- Provides a practical framework for improving genomic prediction and selection across diverse environments.

**TABLE 1:** Total number of environments tested and their mean and median values across all four traits for 3200 wheat breeding lines from 2017 to 2023.

Trait	Environment tested	Min. number of genotype	Max. number of genotype	Mean genotype number	Median genotype number
Yield (kg/ha)	25	8	419	170.2	153
Test weight (kg/m^3^)	25	8	478	184.8	153
Heading date (Jul)	11	8	648	268.6	308
Plant height (cm)	9	8	145	55.7	69

### 2.2 Genomic data

DNA extraction, genotype‐by‐sequencing (GBS) library preparation and SNP calling were conducted as previously described by Winn et al. (2023) at the USDA‐ARS Eastern Regional Small Grains Genotyping Laboratory. Leaf tissue was collected from each line at the four‐leaf stage, and the DNA was extracted using sbeadex plant maxi kits (LGC Genomics) according to the manufacture's protocol. GBS was performed as described by Poland et al. (2012). The libraries were sequenced at 192 plex densities and processed on an Illumnia HiSeq 2500 platform. SNP discovery from the raw sequencing data was performed using the Tassel 5 GBS v2 pipeline version 5.2.35 (Glaubitz et al., 2014).

SNP data were filtered by removing genotypes with 85% or more missing data, minor allele frequencies of 5% or lower, and SNPs with heterozygosity call frequency of 10% or higher using TASSEL v5. Post filtering, SNP imputation was performed using Beagle version 5.2 (Browning & Browning, 2016), which has been shown by Ward et al. (2021) to have imputation accuracy greater than 95% in wheat.

### 2.3 Climate data

Daily weather variables including minimum and maximum temperature, precipitation, and relative humidity were obtained from the NASA POWER (Sparks, 2018) database. Daylength (photoperiod) in hours was calculated from the latitude and day of year using a standard trigonometric approach implemented in R with lubridate (Grolemund & Wickham, 2011). The time interval for a year started from 1 October to 1 September of the following year (e.g., data for 2022 consist of dates between October 1, 2022 and August 31, 2023). Data were then averaged by month so that the total number of features per environment was the product of the five weather variables and 11 months, resulting in 55 weather features per environment. The resulting environmental covariate matrix corresponds to matrix (O), and all environmental covariates were centered and scaled prior to analysis. The geographic coordinates of all sites from which weather data were extracted are presented in Table S1.

### 2.4 Statistical analyses of phenotypic data

Phenotypic best linear unbiased estimates (BLUEs), which represent values adjusted for the unbalanced experimental design and varying replication levels for downstream modeling, were obtained for all traits—grain yield, test weight, heading date, and plant height using ASReml‐R v4.2 (Butler et al., 2023).

Each phenotypic trait for the genotype *i*, in the replication *j*, within the environment *e* was modeled as follows:

$$y_{ij(e)}=\mu_{e}+g_{i(e)}+r_{j(e)}+\varepsilon_{ij\left(e\right)}$$

where *y_ij_* is the observed phenotypic value of the *i*th genotype in the *j*th replication of the *e*th environment, *µ_e_* is the fixed mean effect of environment (*e*), *g_i_*_(_ *_e_*_)_ is the fixed genotype effect of the *i*th genotype in the *e*th environment, *r_j_*_(_ *_e_*_)_ is the random replication effect within the environment (*e*), and *ε_ij_*_(_ *_e_*_)_ is the residual which is assumed to be independently and identically distributed (IID).

Broad sense heritability (*H* ^2^) was estimated using raw data separately for each environment across all traits using the linear mixed model described above, but with genotype and replication effect specified as random. Broad sense heritability (*H* ^2^) was calculated within each environment as follows:

$$\;H^{2}=\frac{\;\sigma_{G\;}^{2}}{\left(\sigma_{G}^{2}+\frac{\sigma_{\varepsilon }^{2}}{r}\right)}$$

where $\sigma_{G}^{2}$ and $\sigma_{\varepsilon }^{2}$ correspond to variance due to genotypic and residual effects and *r* represents the number of replications.

### 2.5 Prediction models

Five different models were used to predict the performance of all four phenotypic traits based on the foundational model structure described by Jarquín et al. (2014). BLUEs obtained from phenotypic traits served as the input of all these models and all factors in the model were treated as random effects. A schematic diagram of all five models is presented in Figure 2.

**FIGURE 2:**
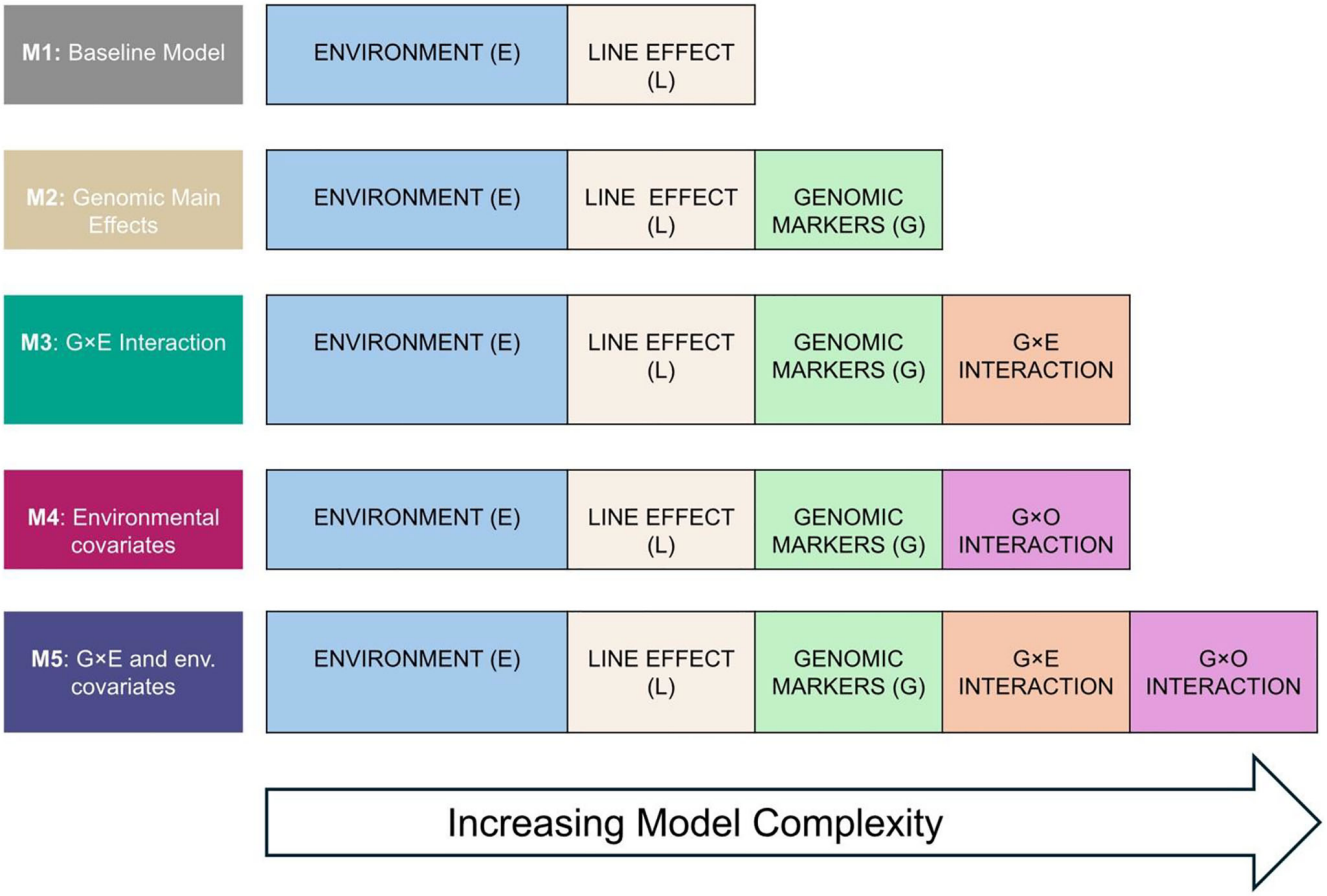
Schematics of all five different prediction models.

#### 2.5.1 M1: Baseline model (*E* + *L*)

Model M1, also called baseline model, integrates main effects for both environment (*E*) and line (*L*) to predict traits and is described as follows:

$$y_{ij}=\mu +E_{i}+L_{j}+\varepsilon_{ij}$$

where the response variable *y_ij_* is the observed phenotypic value of the *j*th line evaluated in the *i*th environment. The model contains overall mean (*μ*), random *i*th environmental effects (*E_i_*), and *j*th line effect (*L_j_*) assuming:

- *E_i_* ∼ *N*(0, *σ* ^2^ *_E_*), where *σ* ^2^ *_E_* is the variance component of environment,
- *L_j_* ∼ *N*(0, *σ* ^2^ *_L_*), where *σ* ^2^ *_L_* is the variance component of line, and
- *ε_ij_* ∼ *N*(0, $\sigma_{\varepsilon }^{2}$), where $\sigma_{\varepsilon }^{2}$ is the residual variance.

As both *E* = {*E_i_*} and *L* = {*L_j_*} assume independence across different levels of environment and line, this assumption makes the model unable to borrow information from related environments/genotypes. In practice, if a line or environment has not been observed, then this model becomes null as it just predicts the average (*μ*). Hence, it was used as a negative control to quantify the differences between CV2 (random cross‐validation) and CV1 (genotype constrained cross validation).

#### 2.5.2 M2: *E* + *L* + *G*

In the M2 model, the SNP markers were introduced in the baseline model as follows:

$$y_{\mathrm{ij}}=\mu +E_{i}+L_{j}+g_{j}+\varepsilon_{\mathrm{ij}}$$

where *g_j_* follows the multivariate normal distribution with **g** = {*g_j_*} ∼ *N*(**0**, ***G**σ* ^2^_g_). The covariance matrix Cov(**g**) = ***G**σ* ^2^_g_, where ***G*** = $\frac{{\mathrm{XX}}^{\prime }}{p}$ denotes the genomic relationship matrix, *X* is the centered and standardized genotype matrix to *p* SNP markers, and *σ* ^2^_g_ is the corresponding genomic variance.

The ***G*** term in the M2 model accounts for genotypic effects where SNP marker information is used to establish relationships between each pair of genotypes. This allows borrowing of information such that if a given genotype performs well, then a related genotype is expected to perform in a similar manner.

#### 2.5.3 M3: *E* + *L* + *G* + *GE*

Model M3 extends model M2 and includes a G × E interaction component.

$$y_{\mathrm{ij}}=\mu +E_{i}+L_{j}+g_{j}+gE_{\mathrm{ij}}+\varepsilon_{\mathrm{ij}}$$

The model is similar to M2 with the addition of *gE_ij_* component, which denotes interaction of the *i*th genotype with the *j*th environment with

$$gE=\left\{gE_{ij}\right\}∼N\left(0,{\left(Z_{g}{GZ}_{g}^{\prime }\right)}^{\circ }\left(Z_{E}Z_{E}\right)\sigma_{gE}^{2}\right),$$

where ***Z***_g_ and ***Z***_*E*_ are the incidence matrices for lines and environment, respectively. *σ* ^2^_g_ *_E_* is the corresponding variance component of g*E_ij_*. The sign “°” is the Hadamard product between two matrices that denotes elementwise product between two matrices.

The GE term accounts for the interaction between genotype and environment allowing different behaviors of the same genotype in different environments. This enables model adaptation and superiority.

#### 2.5.4 M4: *E* + *L* + *G* + *GO*

In model M3, it was assumed that environments were independent for GE interaction purposes, that is, although two related genotypes will perform similarly in a given environment, it is impossible to predict whether two environments will show the same patterns. The *GO* term uses weather data to define "mega‐environments," where G × E patterns are assumed to be similar. Model M4 is as follows:

$$y_{\mathrm{ij}}=\mu +E_{i}+L_{j}+g_{j}+gW_{\mathrm{ij}}+\varepsilon_{\mathrm{ij}}$$

where **g** ***W*** = {g*W_ij_*} ∼ *N*(**0**, (***Z***_g_ ***GZ*** *′*_g_) ° ($Z_{E}{\mathrm{OZ}}_{E}^{\prime }$) *σ* ^2^_g*O*_) where g*W_ij_* is the effect of *j*th genotype at *i*th environment, *W* is the matrix of *q* weather covariates, and ***O*** = $\frac{{\mathrm{WW}}^{\prime }}{q}$ is the matrix of environmental similarities between pairs of environments.

In this equation $Z_{E}{\mathrm{OZ}}_{E}$ replaces $Z_{E}Z_{E}^{\prime }$ as a measure of similarity between environments based on covariates. *σ^2^_gO_* is the variance of *GO* interaction.

As we were interested in within‐environment variation, the ***O*** term is not included in this model because it is a main effect that applies to every phenotype in a given environment and therefore captures only the average effect of weather across all genotypes.

#### 2.5.5 M5: *E* + *L* + *G* + *GE *+ *GO*

Model M5 integrates all different terms introduced from M1 to M4 and is the most complex model among all models.

$$y_{\mathrm{ijk}}=\mu +E_{i}+L_{j}+g_{j}+gE_{\mathrm{ij}}+gW_{\mathrm{ij}}+\varepsilon_{\mathrm{ij}}$$

All previously described terms from models M3 and M4 are contained in M5 with both *GE* and *GO* interaction present in this model.

This model combines both *GE* and *GO* to approximate the ranking within a given environment under the assumption of environmental relatedness according to weather data (*GO* term) and the assumption of unrelatedness (GE term). While GE captures how genotypes react differently to discrete environments, *GO* captures the plasticity of genotypes across environmental gradients.

### 2.6 Variance component estimation

Variance components were estimated to partition genetic background, marker‐based genetic effects, and environment and weather‐dependent genetic responses while accounting for residual variability.

The total phenotypic variance was defined as follows:

$${\sigma^{2}}_{P}={\sigma^{2}}_{L}+{\sigma^{2}}_{G}+{\sigma^{2}}_{\mathrm{GE}}+{\sigma^{2}}_{\mathrm{GO}}+{\sigma^{2}}_{R}$$

where *σ* ^2^_*L*_ is the line variance, *σ* ^2^_*G*_ is the marker‐based genomic variance, *σ* ^2^_*GE*_ is the G × E interaction variance, *σ* ^2^_*GO*_ is the genotype × weather covariate interaction variance and *σ* ^2^_R_ is the residual variance.

### 2.7 Cross validation

To evaluate the accuracy of all five implemented models, cross‐validation (CV) schemes were implemented based on the strategy described by Jarquín et al. (2017). To validate the models, the phenotypic dataset for all four traits was split into different training and testing sets using *k*‐fold CV schemes.

#### 2.7.1 CV2: Prediction of lines in incomplete trials

This cross‐validation scheme is used to predict known genotype performance in environments where some data are missing for certain genotypes. Lines were randomly assigned to five cross‐validation folds, and the process was repeated over 10 independent random runs (50 total evaluation runs). In each fold, 80% (or four folds) of phenotypic data were used for training. For each fold, the model was fitted using a total of 12,000 iterations with a burn‐in period of 2000. Predictions were then obtained for the 20% of observations held out for validation. Additionally, for training and testing split, it was ensured that the training set may include observations of the same genotype in other environments (e.g., Line 1 in Env2 or Line 2 in Env1), but never the target genotype in the target environment (e.g., Line1 in Env1) since that would constitute data leak.

#### 2.7.2 CV1: Prediction of new lines in known environments

CV1 scheme predicts new lines that have not been observed in previously tested environments. Since the folds in CV1 are constrained by genotypes, whole genotypes were held out during CV rather than individual observations. Thus, CV1 scheme evaluates if the model can predict new genotypes based on genetically related genotypes it has already seen.

Data for a given genotype were held together in the same folds such that none of them are used for training when that genotype is being predicted. Data from CV1 were also assigned to fivefolds with 80%–20% training‐testing split and the process was repeated 10 times. Following cross‐validation, correlation between observed and predicted values within the same environments was calculated.

#### 2.7.3 CV0: Prediction of lines in untested environments

The CV0 scheme aims to predict the performance of lines in previously untested environments. While CV1 was constrained by genotypes, CV0 is constrained by a new environment. Thus, CV0 aims to predict genotype performance in those environments that were not used in model training but were previously observed in other environments. In the CV0 scheme, the testing environment was completely left out, and the model was trained using data from all the remaining environments. This trained model was then used to predict line performance in the earlier excluded environments. Since environments were excluded one at a time, CV0 does not involve random partitioning like CV1 or CV2 and thus was performed only once.

#### 2.7.4 CV00: Prediction of untested lines in untested environments

The CV00 scheme combines both CV1 and CV0 restrictions and aims to predict new lines in new environments. In the CV00 strategy, the model was trained using untested lines that are predicted in environments where they have not been evaluated using information from different sets of lines tested in other environments. The model was trained using data from the remaining genotypes evaluated in other environments. Since the CV00 scheme involves systematic exclusion of both genotypes and environments rather than random sampling, CV00 was performed only once. Figure S2 summarizes the cross‐validation schemes implemented in this study.

### 2.8 Prediction accuracy

The prediction accuracy of all five models (M1–M5) tested across four CV schemes was evaluated by calculating the Pearson correlation coefficient (*r*) between the observed and predicted values within each environment. The values from different environments were averaged using the number of observations that led to the correlation as the weighting factor.

Rather than calculating a simple arithmetic mean across environments, the overall global prediction accuracy was reported using an inverse‐variance weighting approach. This method accounts for the heterogeneity in sample sizes and varying degree of data volatility across environments (Borenstein et al., 2021; Tiezzi et al., 2017). This weighting prevents environments with small sample sizes or high sampling error from biasing the global average.

The weighted prediction accuracy as proposed by Tiezzi et al. (2017) was calculated as follows:

$$\;r_{w}=\frac{\left(\sum_{\left(i=1\right)}^{n_{E}}\frac{r_{i}}{V\left(r_{i}\right)}\right)}{\left(\sum_{\left(i=1\right)}^{n_{E}}\frac{1}{V\left(r_{i}\right)}\right)}$$

where $V\left(r_{i}\right)=\frac{1-r_{i}^{2}}{n_{i}-2}$, *r_i_* represents the Pearson correlation between the observed and predicted values at the *i*th environment, *V*(*r_i_*) corresponds to the sampling variance, *n_i_* corresponds to the number of observations in the current environment and *n* *_E_* is the number of environments.

## 3 RESULTS

### 3.1 Genotyping

A total of 40,850 high‐quality SNP markers were retained for downstream modeling after SNP filtering across 3200 wheat lines. SNP were mapped across an estimated physical genome length of 14,053.81 MB and the final marker panel gave a high‐resolution genome‐wide average density of 2.91 SNPs/MB (Figure S3). Marker density shifted across the genomes, following a typical pattern for wheat. The highest concentrations of SNPs were at the outer tips of the chromosomes while the centromere region had fewer markers. This genome‐wide coverage served as a reliable genetic baseline ensuring we had the resolution needed to build accurate relationship matrices for our prediction models (M2–M5).

### 3.2 Phenotyping

The phenotypic dataset showed that grain yield and test weight had the highest number of testing locations (25) while plant height had the fewest followed by heading date (Table 1). This is typical in a public breeding program where yield‐related traits are prioritized more over agronomic traits because of limited resources for gathering data on multiple traits. However, across all 25 environments, heading date had the maximum number of genotypes (648) followed by test weight and yield, likely because agronomic traits like heading time are easier and quicker to measure when assessed across multiple environments. Table 1 summarizes mean and median genotype numbers for all four traits measured across testing environments.

Due to the low level of genotypes overlapping, it is challenging to compute genetic correlations between environments for a given trait. However, we can pair observations of the same genotypes for different traits. In doing so, significant correlation was found for yield–heading date and test weight–plant height pairs (Figure S4).

Heritability (*H* ^2^) varied across environments and traits with heading date showing the highest *H* ^2^ value ranging from 0.35 to 0.95, while test weight had the highest in the CLAYTON 2022 environment reflecting environmental influence on expression of phenotype. *H* ^2^ for yield ranged between 0.33 and 0.87 (Table S2).

### 3.3 Relationship among weather variables

Five weather variables obtained from NASA POWER were used to construct a kinship matrix, which was then clustered using agglomerative hierarchical clustering (Figure 3). Strong associations of weather variables were observed within the same year compared with across years regardless of locations. This pattern is expected as weather conditions within a given year tend to be more similar than those of past or future years making weather an important factor to consider in breeding cycle decisions. Furthermore, principal component analysis was done where environments were clustered by year. Two environments from the same year were more similar than two environments from the same location across different years. The first two principal components (PC) explained 41.2% and 28.5% of the variance, respectively. A total of 85% and 97% of the variance was explained with 3 and 5 PCs, respectively (Figure S5).

**FIGURE 3:**
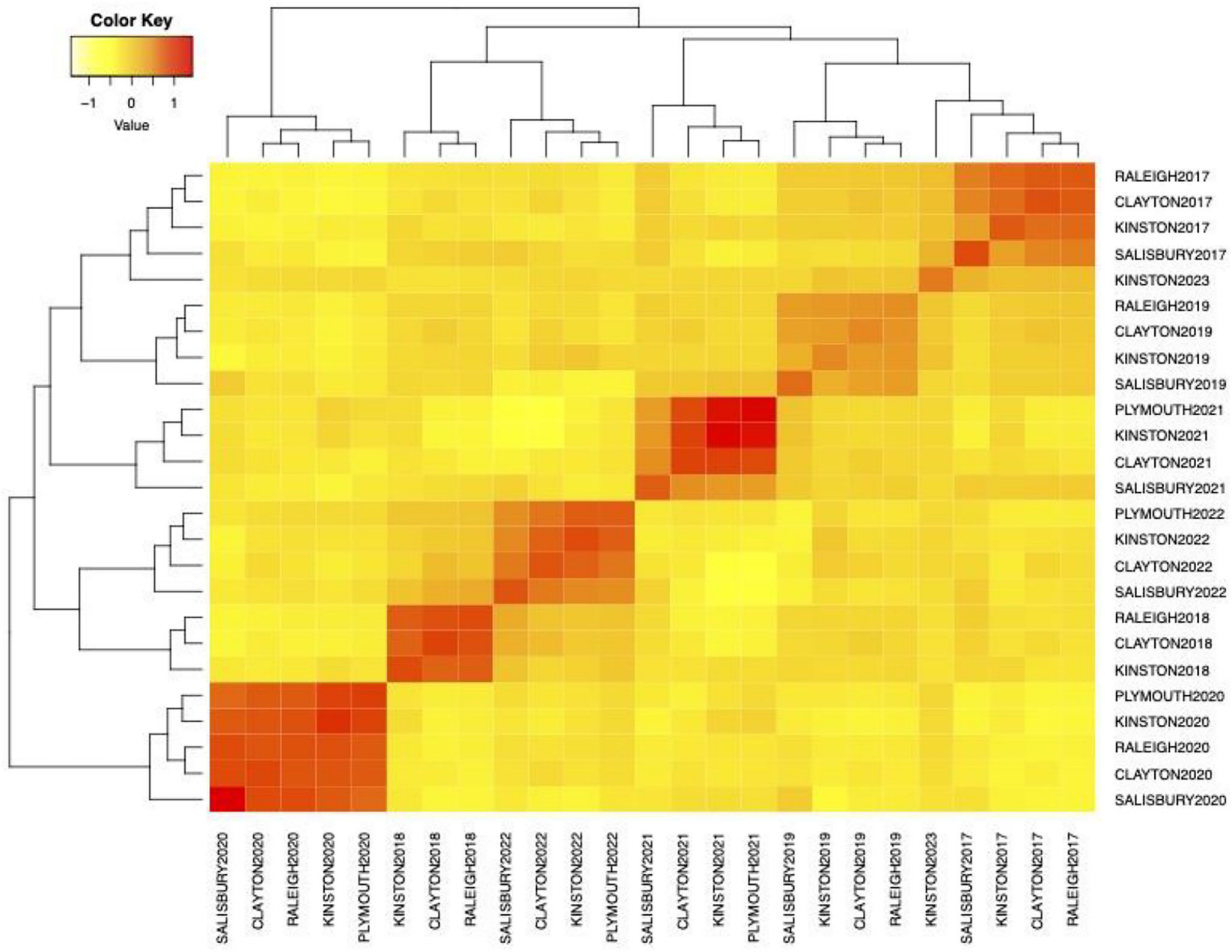
Environment variance‐covariance matrix derived from weather covariables and clustered using the agglomerative hierarchical clustering algorithm. Distinct clusters highlighted in red squares show that weather data from different environments within the same year were more closely associated than data from different years, regardless of location.

### 3.4 Variance component analysis

Phenotypic variance (before cross‐validation) was used to quantify the relative contribution of each term (E, G, and O) and their interactions (G × E and G × O) across all four traits. Variance partitioning was calculated as the percentage of variance explained by each variance component and is presented in Figure 4. The percentage of variance explained by line (varL) in the baseline model (M1) can be seen as broad‐sense heritability while the variance due to marker (varG) is more aligned with narrow‐sense heritability. Residual variance (varR) represented the proportion of variance that could not be explained by any of the terms in the model. The complete variance partitioning is included in Table S3.

**FIGURE 4:**
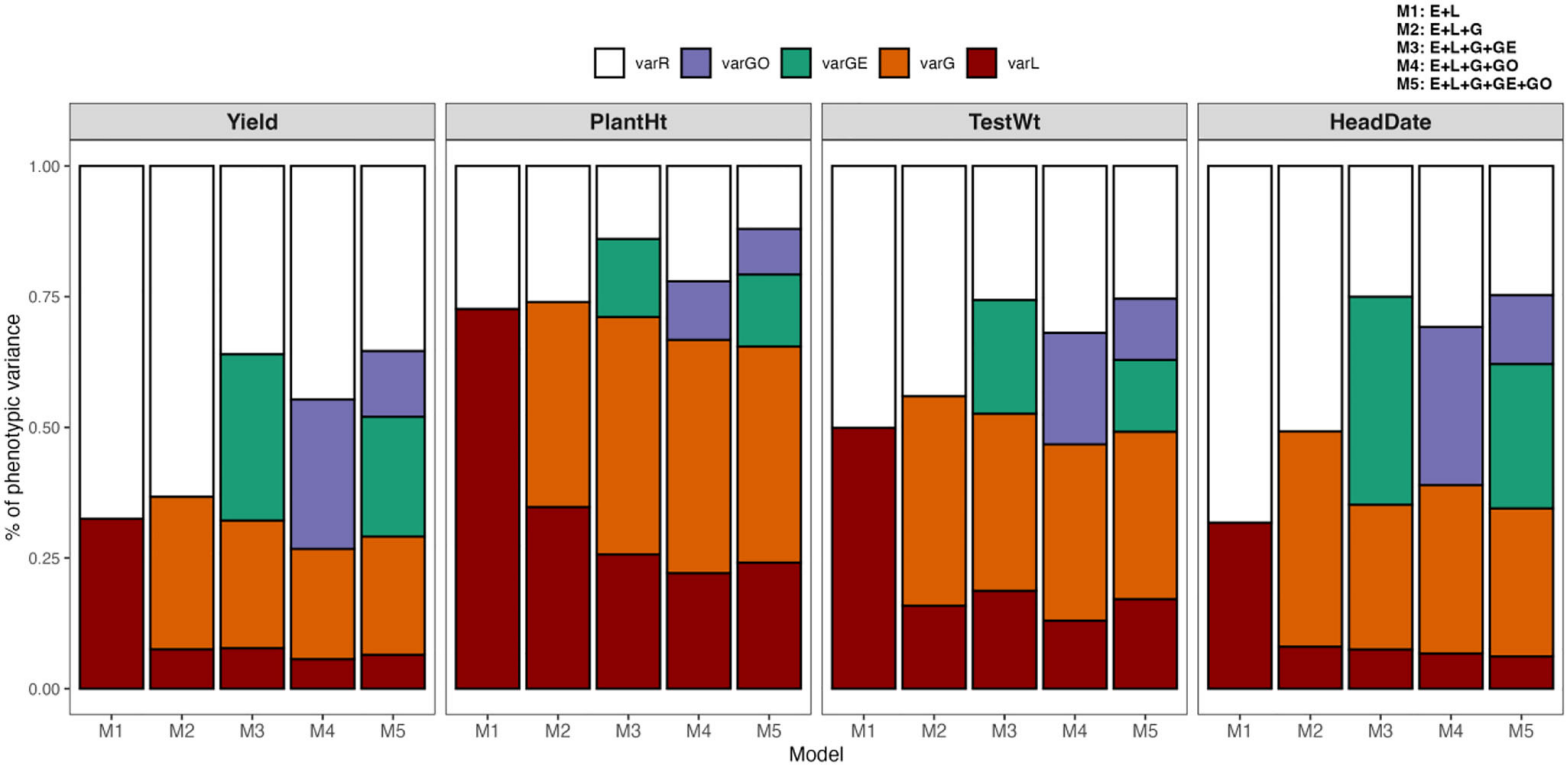
Partitioning of phenotypic variance measured across all four traits. The percentage of variance explained by each term other than environment is shown on the y‐axis. Models are denoted on the x‐axis, and each trait is represented in a different set of columns. varL represents variance due to line, varG represents variance due to single nucleotide polymorphism (SNP) marker, varR represents residual variance, varGE and varGO represent variance due to interaction terms G × E and G × O, respectively.

In the baseline model M1 (*E* + *L*), phenotypic variance was partitioned into varL and varR. For three traits—yield, test weight, and heading date—the largest proportion of phenotypic variance was due to varR indicating substantial unexplained variability beyond genetic differences among lines (Figure 4). However, for plant height, varL exceeded varR indicating strong genetic control across environments. With the addition of G in the second model (M2), the sum of *L* + *G* could explain slightly more variability than *L* alone in the baseline model. Subsequently, as additional term (G × E) was added to the M3 model, the G × E interaction variance (varGE) explained more variance by further reducing residual variance. In the fifth model (M5) where all terms were combined, residual variances were like those of the M3 model, suggesting that GE alone captured G × E interaction variance and the GO term might be somehow redundant.

### 3.5 Prediction models evaluation using cross‐validation

The prediction accuracies for the four evaluated traits, calculated as described previously, are represented in Figure 5 and Table S4.

**FIGURE 5:**
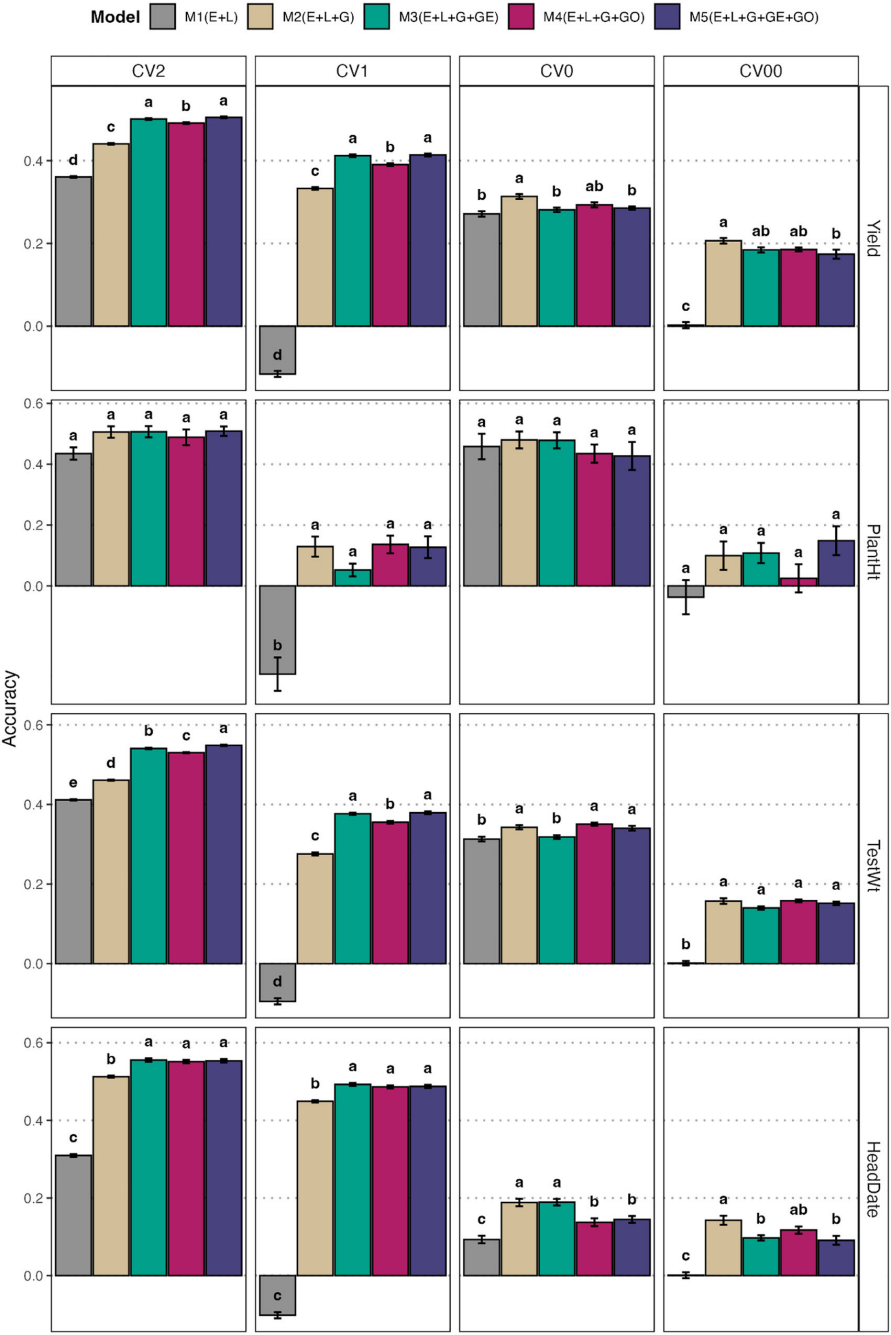
Accuracy achieved by all five models (M1–M5) under various cross‐validation scenarios (columns) across four traits (rows). In each grid, accuracy is indicated on the y‐axis, while models are represented on the x‐axis, corresponding to their respective colors. Significant letters were assigned using Tukey‐adjusted pairwise comparisons of model mean (*α* = 0.05), accounting for variation among repeated CV runs. Different lowercase letters indicate statistically significant differences among models within each CV scheme and trait.

#### 3.5.1 Predicting lines in incomplete trials (CV2)

The most complex model M5 achieved the highest accuracy for yield (0.51), plant height (0.51), and test weight (0.55) in the incomplete trial predictions (CV2). However, adding the weather covariate (GO) in the M5 model only provided a significant accuracy boost for test weight when compared to using GE alone (M3 model). For yield, M5 model performance was not significantly different from the M3 model (0.50). Similarly, while model M3 achieved highest accuracy for heading date (0.56) it did not significantly differ from M4 or M5. Notably for plant height, no significant differences in accuracy were observed across any of the five evaluated models.

#### 3.5.2 Predicting new lines (CV1)

Although model M5 reached the highest accuracy when predicting yield (0.41) and test weight (0.38) for new lines in known environments, adding weather data (GO) did not provide a significant improvement over using GE interactions (M3) alone. Heading date followed a similar pattern with the highest accuracy of 0.49 for M3 but no significant differences among M3, M4, and M5 models. Prediction accuracies were notably lowest for plant height. While model M4 achieved the highest accuracy for height (0.14), its performance was statistically similar to M2, M3, and M5 models.

#### 3.5.3 Predicting new environment (CV0)

Based on the prediction of a new environment from known genotype information using the CV0 scheme, adding complex interaction terms (GE and GO) yielded minimal improvements. Prediction accuracies varied notably among the four traits. Plant height showed the highest accuracy (0.47 for both the M2 and M3 models), with no significant differences detected among any of the five models. Conversely, heading date showed the lowest overall accuracies, though the M3 model (0.19) incorporating GE significantly outperformed the M4 and M5 models. For yield, the M2 achieved the highest significant accuracy (0.31) compared to the M3 and M5 models. Although the M4 model achieved the highest accuracy for test weight (0.35) and significantly outperformed the M3 model, its predictive performance remained statistically similar to both the M2 and M5 models.

#### 3.5.4 Predicting new lines in new environment (CV00)

Predicting new genotypes in unobserved environments using the CV00 scheme showed consistently low prediction accuracies (0.21 and below) across all four traits. Incorporating complex interaction terms (GE and GO) largely failed to improve accuracy over the M2 model. Yield prediction for the M2 model reached the highest (0.21) which significantly outperformed the more complex M5 model. Plant height showed no significant differences among any of the five models. The M2 model achieved the highest accuracy for heading date (0.14) and significantly outperformed the M3 and M5 models. Test weight showed the highest accuracy (0.16) with the M4 model but remained statistically similar to the M2, M3, and M5 models.

#### 3.5.5 Mean squared error

Following prediction accuracy, mean squared error (MSE) was computed as the average of the squared differences between observed and predicted values. The MSE values were similar within the pairs CV2–CV1 and CV0–CV00 with lower MSE observed for CV2–CV1 than for CV0–CV00 across all traits (Figure S6). The general trend for MSE in CV2–CV1 showed that models including an interaction term (GE, GO, or both) did better in predicting yield test weight, and heading date. Under the CV2 scheme, the lowest MSE was observed for yield (70.8), plant height (3.4), and test weight (1.7) in the M5 models that included both GE and GO interaction terms, while the lowest MSE for heading date (6.0) was observed in the M3 model. For CV1, the M3 model had the lowest MSE for yield (78.9) and heading date (6.7), whereas the lowest MSE for plant height (5.3) and test weight (2.1) were achieved by the M2 and M3 models, respectively.

For the CV00 and CV0 schemes, model performance was primarily driven by the M2 and M4 models. Under CV00, the lowest MSE was observed for yield (262.7) and heading date (65.3) in the M2 model, while the M4 model achieved the lowest MSE for plant height (16.6) and test weight (7.2). For CV0, the M4 model again achieved the lowest MSE for plant height (14.1) and test weight (6.5). In contrast, the lowest MSE for yield (249.1) was observed in the M2 model and the lowest MSE for heading date (64.6) was achieved by the M1 model.

## 4 DISCUSSION

GS has revolutionized wheat breeding by enabling the prediction of breeding values of new and untested lines, allowing breeders to make early selection decisions, shorten breeding cycles, and optimize resource allocation (Bandillo et al., 2023; Bhat et al., 2016; Jannink et al., 2010; Juliana et al., 2018). In the North Carolina wheat growing regions where the environment is highly heterogeneous, genotypes often respond differently across environments, leading to strong G × E interactions that challenge the assumption of stable genotypic performance. Traditional baseline models (M1) that rely solely on main additive genetic effects fail to account for the phenotypic plasticity of lines in such changing environmental conditions.

To address this limitation, we adopted a RNM framework. Unlike conventional approaches that treat environments a discrete category, our RNM incorporates EC, allowing the estimation of genotypic‐specific responses to environmental gradients (G × O interactions). Although standard G × E models (M3) improve prediction by borrowing information across genetically related lines, the integration of weather‐based covariates in RNMs (M4 and M5) further allows models to borrow information across environments with similar climatic conditions. This EC integration is particularly important for complex trait like grain yield where performance is strongly influenced by environmental variability.

In genomic prediction breeding literature, incomplete field trials (CV2) and the prediction of untested lines (CV1) are the most common scenarios reported (Burgueño et al., 2012). In our study, the full reaction norm framework (M5), which incorporates both G × E and G × O interactions, gave the highest prediction accuracies in CV2 for yield and test weight outperforming the baseline model (M1) by up to 40% and the M2 model by up to 25%. This superior performance of M5 in CV2 is largely due to model's ability to represent environmental variation using biologically meaningful weather variables, such as temperature and precipitation during key crop growth stages, instead of treating each environment as independent factors. By modeling these environmental variables as continuous predictors, M5 captures genotype‐specific responses to dynamic stressors such as heat. Jarquín et al. (2014, 2017) demonstrated that coupling ECs with high‐density markers significantly boosts the model accuracy for grain yield. In contrast, our plant height trait showed no significant variation in prediction across M2–M5 models.

Interestingly, although the M5 model excelled for yield and test weight in CV1, adding G × O interactions provided marginal improvements over the simpler G × E model (M3) for heading date. In fact, across both CV1 and CV2, M3 consistently achieved the highest accuracy for heading date, improving performance by up to 10% compared to M2. This suggests that the high‐resolution ECs might have introduced model complexity without providing meaningful gain for this trait.

Our results suggest that the value of increasing model complexity might depend on the genetic architecture of the trait being predicted. Predicting seed yield clearly benefited from the RNM models that incorporated ECs (M5), whereas heading date and plant height showed negligible improvements as model complexity increased. This difference likely reflects the distinct genetic basis of the traits. Phenological developments and plant stature in wheat are controlled primarily by few major‐effect genes whose effects are driven by broad environmental factors including photoperiod and thermal time (Guedira et al., 2016; Lozada et al., 2021). In durum wheat, Puglisi et al. (2026) showed that incorporating functional markers (through target‐gene matrices, G_2_) for major genes was sufficient to achieve high prediction accuracy for heading date and plant height. Although adding detailed environmental information through RNM offered little additional benefit for single‐trait prediction, prediction accuracy increased substantially when these traits were analyzed using multi‐trait multi‐environment models.

Forward‐predicting line performance in a completely unobserved environment (CV0) or predicting entirely new lines in new environments (CV00) remains the most challenging scenario in prediction. In our study, the M2 model which lacks the complex G × E and G × O interaction terms unexpectedly outperformed the more advance RNMs (M3–M5). This shift in model performance highlights a critical bias‐variance tradeoff in genomic prediction. While M4–M5 models are highly effective at explaining variance within the training data (CV1/CV2), they are highly susceptible to over‐parameterization when applied to naive environments. Because CV0 and CV00 lack direct phenotypic information for the target environments, the specific ECs‐genotype interaction patterns learned in the training set may not transfer reliably, resulting in reduced prediction accuracy in unobserved environments. Ankamah‐Yeboah et al. (2020) and Jarquín et al. (2017) also reported this limitation in covariate transferability under CV00 where prediction accuracy often was below 0.20.

The limitations observed in our CV0/CV00 predictions contextualize recent methodological advancements in enviromics in plant breeding (Costa‐Neto et al., 2021). For example, while our study relied exclusively on dynamic weather covariates, recent applications of RNMs that have utilized other environmental covariates, such as soil properties, have shown improved transferability across environments (Canella Vieira et al., 2022). Furthermore, advanced framework utilizing joint genomic regression analysis has shown potential for reducing environmental noise and identifying latent environmental features that improve prediction accuracy (Brault et al., 2025; Zhu et al., 2026). Future research in North Carolina wheat regions could potentially benefit from integrating these approaches to better capture G × E variations and improve prediction accuracy across diverse environments.

Despite the challenges in forward prediction, the strong performance of M5 under the CV2 scenario has important implication for applied wheat breeding. The ability to predict in incomplete trial scenario with greater accuracy means that breeders can implement more efficient sparse testing designs. By selectively planting only a subset of genotypes in each environment and predicting the remainder using EC‐integrated RNMs, breeding programs could expand testing across variable North Carolina wheat growing regions without proportionally increasing phenotyping costs.

## 5 CONCLUSION

North Carolina and other parts of the southeastern United States experience highly variable wheat‐growing conditions due to spatial and temporal differences in weather. Prediction models that integrate the main and interaction effects of high‐dimensional genomic markers with weather‐based environmental responses, such as our M5 model, substantially improve prediction accuracy. However, the benefit of modeling G × E alone (M3 model) is trait‐dependent and appears greatest for traits with repeatable interaction patterns across environments, even without weather covariates. The relatively low prediction accuracies observed when predicting new lines in new environments will likely indicate that further gains will require broader multi‐environment trial coverage and more informative environmental characterization, including growth stage‐specific weather variables.

## AUTHOR CONTRIBUTIONS

**Shailesh Raj Acharya**: Data curation; formal analysis; investigation; methodology; writing—original draft; writing—review and editing. **Julian Garcia‐Abadillo**: Formal analysis; writing—review and editing. **Jeanette Lyerly**: Data curation; writing—review and editing. **Gina Brown‐Guedira**: Writing—review and editing. **Diego Jarquin**: Formal analysis; writing—review and editing. **Nonoy Bandillo**: Conceptualization; funding acquisition; investigation; methodology; supervision; writing—review and editing.

## CONFLICT OF INTEREST STATEMENT

The authors declare no conflicts of interest.

## Supporting information



Supplementary Material

## Data Availability

The R code and associated workflow scripts are archived in Zenodo (https://doi.org/10.5281/zenodo.22031426). Dataset is available from the corresponding author upon reasonable request, subject to any applicable institutional or data‐use requirements.

## References

[tpg270332-bib-0001] Ankamah‐Yeboah, T. , Janss, L. L. , Jensen, J. D. , Hjortshøj, R. L. , & Rasmussen, S. K. (2020). Genomic selection using pedigree and marker‐by‐environment interaction for barley seed quality traits from two commercial breeding programs. Frontiers in Plant Science, 11, 539. 10.3389/fpls.2020.00539 PMC7227446 32457780

[tpg270332-bib-0002] Atanda, S. A. , & Bandillo, N. (2024). Genomic‐inferred cross‐selection methods for multi‐trait improvement in a recurrent selection breeding program. Plant Methods, 20(1), 133. 10.1186/s13007-024-01258-4 PMC11367796 39218896

[tpg270332-bib-0003] Bandillo, N. B. , Jarquin, D. , Posadas, L. G. , Lorenz, A. J. , & Graef, G. L. (2023). Genomic selection performs as effectively as phenotypic selection for increasing seed yield in soybean. The Plant Genome, 16(1), e20285. 10.1002/tpg2.20285 PMC12807388 36447395

[tpg270332-bib-0004] Bhat, J. A. , Ali, S. , Salgotra, R. K. , Mir, Z. A. , Dutta, S. , Jadon, V. , Tyagi, A. , Mushtaq, M. , Jain, N. , Singh, P. K. , Singh, G. P. , & Prabhu, K. V. (2016). Genomic selection in the era of next generation sequencing for complex traits in plant breeding. Frontiers in Genetics, 7, 221. 10.3389/fgene.2016.00221 PMC5186759 28083016

[tpg270332-bib-0005] Borenstein, M. , Hedges, L. V. , Higgins, J. P. T. , & Rothstein, H. R. (2021). Introduction to meta‐analysis. John Wiley & Sons.

[tpg270332-bib-0006] Brault, C. , Conley, E. J. , Read, A. C. , Green, A. J. , Glover, K. D. , Cook, J. P. , Gill, H. S. , Fiedler, J. D. , & Anderson, J. A. (2025). Improving genomic prediction for plant disease using environmental covariates. Plant Methods, 21(1), 114. 10.1186/s13007-025-01418-0 PMC12366029 40836294

[tpg270332-bib-0007] Browning, B. L. , & Browning, S. R. (2016). Genotype imputation with millions of reference samples. The American Journal of Human Genetics, 98(1), 116–126. 10.1016/j.ajhg.2015.11.020 PMC4716681 26748515

[tpg270332-bib-0008] Burgueño, J. , de los Campos, G. , Weigel, K. , & Crossa, J. (2012). Genomic prediction of breeding values when modeling genotype × environment interaction using pedigree and dense molecular markers. Crop Science, 52(2), 707–719. 10.2135/cropsci2011.06.0299

[tpg270332-bib-0009] Butler, D. G. , Cullis, B. R. , Gilmour, A. R. , Gogel, B. G. , & Thompson, R. (2023). ASReml‐R reference manual version 4.2. VSN International Ltd.

[tpg270332-bib-0010] Canella Vieira, C. , Persa, R. , Chen, P. , & Jarquin, D. (2022). Incorporation of soil‐derived covariates in progeny testing and line selection to enhance genomic prediction accuracy in soybean breeding. Frontiers in Genetics, 13, 905824. 10.3389/fgene.2022.905824 PMC9493273 36159995

[tpg270332-bib-0011] Costa‐Neto, G. , Crossa, J. , & Fritsche‐Neto, R. (2021). Enviromic assembly increases accuracy and reduces costs of the genomic prediction for yield plasticity in maize. Frontiers in Plant Science, 12, 717552. 10.3389/fpls.2021.717552 PMC8529011 34691099

[tpg270332-bib-0012] Crossa, J. , Campos, G. D. L. , Pérez, P. , Gianola, D. , Burgueño, J. , Araus, J. L. , Makumbi, D. , Singh, R. P. , Dreisigacker, S. , Yan, J. , Arief, V. , Banziger, M. , & Braun, H.‐J. (2010). Prediction of genetic values of quantitative traits in plant breeding using pedigree and molecular markers. Genetics, 186(2), 713–724. 10.1534/genetics.110.118521 PMC2954475 20813882

[tpg270332-bib-0013] Crossa, J. , Pérez‐Rodríguez, P. , Cuevas, J. , Montesinos‐López, O. , Jarquín, D. , de los Campos, G. , Burgueño, J. , González‐Camacho, J. M. , Pérez‐Elizalde, S. , Beyene, Y. , Dreisigacker, S. , Singh, R. , Zhang, X. , Gowda, M. , Roorkiwal, M. , Rutkoski, J. , & Varshney, R. K. (2017). Genomic selection in plant breeding: Methods, models, and perspectives. Trends in Plant Science, 22(11), 961–975. 10.1016/j.tplants.2017.08.011 28965742

[tpg270332-bib-0014] Glaubitz, J. C. , Casstevens, T. M. , Lu, F. , Harriman, J. , Elshire, R. J. , Sun, Q. , & Buckler, E. S. (2014). TASSEL‐GBS: A high capacity genotyping by sequencing analysis pipeline. PLoS One, 9(2), e90346. 10.1371/journal.pone.0090346 PMC3938676 24587335

[tpg270332-bib-0015] Grolemund, G. , & Wickham, H. (2011). Dates and times made easy with lubridate. Journal of Statistical Software, 40(3), 1–25. 10.18637/jss.v040.i03

[tpg270332-bib-0016] Guedira, M. , Xiong, M. , Hao, Y. F. , Johnson, J. , Harrison, S. , Marshall, D. , & Brown‐Guedira, G. (2016). Heading date QTL in winter wheat (*Triticum aestivum* L.) coincide with major developmental genes VERNALIZATION1 and PHOTOPERIOD1. PLoS One, 11(5), e0154242. 10.1371/journal.pone.0154242 PMC4862677 27163605

[tpg270332-bib-0017] Jannink, J.‐L. , Lorenz, A. J. , & Iwata, H. (2010). Genomic selection in plant breeding: From theory to practice. Briefings in Functional Genomics, 9(2), 166–177. 10.1093/bfgp/elq001 20156985

[tpg270332-bib-0018] Jarquín, D. , Crossa, J. , Lacaze, X. , Du Cheyron, P. , Daucourt, J. , Lorgeou, J. , Piraux, F. , Guerreiro, L. , Pérez, P. , Calus, M. , Burgueño, J. , & de los Campos, G. (2014). A reaction norm model for genomic selection using high‐dimensional genomic and environmental data. Theoretical and Applied Genetics, 127(3), 595–607. 10.1007/s00122-013-2243-1 PMC3931944 24337101

[tpg270332-bib-0019] Jarquín, D. , Lemes da Silva, C. , Gaynor, R. C. , Poland, J. , Fritz, A. , Howard, R. , Battenfield, S. , & Crossa, J. (2017). Increasing genomic‐enabled prediction accuracy by modeling genotype × environment interactions in Kansas wheat. The Plant Genome, 10(2), 10.3835/plantgenome2016.12.0130 28724062

[tpg270332-bib-0020] Juliana, P. , Singh, R. P. , Poland, J. , Mondal, S. , Crossa, J. , Montesinos‐López, O. A. , Dreisigacker, S. , Pérez‐Rodríguez, P. , Huerta‐Espino, J. , Crespo‐Herrera, L. , & Govindan, V. (2018). Prospects and challenges of applied genomic selection—A new paradigm in breeding for grain yield in bread wheat. The Plant Genome, 11(3), 180017. 10.3835/plantgenome2018.03.0017 PMC7822054 30512048

[tpg270332-bib-0021] Lozada, D. N. , Carter, A. H. , & Mason, R. E. (2021). Unlocking the yield potential of wheat: Influence of major growth habit and adaptation genes. Crop Breeding, Genetics and Genomics, 3(2), e210004. 10.20900/cbgg20210004

[tpg270332-bib-0022] McBreen, J. , Babar, M. A. , Jarquin, D. , Khan, N. , Harrison, S. , DeWitt, N. , Mergoum, M. , Lopez, B. , Boyles, R. , Lyerly, J. , Murphy, J. P. , Shakiba, E. , Sutton, R. , Ibrahim, A. , Howell, K. , Smith, J. H. , Brown‐Guedira, G. , Tiwari, V. , Santantonio, N. , & Van Sanford, D. A. (2025). Enhancing prediction accuracy of grain yield in wheat lines adapted to the southeastern United States through multivariate and multi‐environment genomic prediction models incorporating spectral and thermal information. The Plant Genome, 18(1), e20532. 10.1002/tpg2.20532 PMC11726420 39562415

[tpg270332-bib-0023] Meuwissen, T. H. E. , Hayes, B. J. , & Goddard, M. E. (2001). Prediction of total genetic value using genome‐wide dense marker maps. Genetics, 157(4), 1819–1829. 10.1093/genetics/157.4.1819 PMC1461589 11290733

[tpg270332-bib-0024] Poland, J. A. , Brown, P. J. , Sorrells, M. E. , & Jannink, J.‐L. (2012). Development of high‐density genetic maps for barley and wheat using a novel two‐enzyme genotyping‐by‐sequencing approach. PLoS One, 7(2), e32253. 10.1371/journal.pone.0032253 PMC3289635 22389690

[tpg270332-bib-0025] Puglisi, D. , Crossa, J. , Cuevas, J. , Fania, F. , Afshari‐Behbahanizadeh, S. , Vitale, P. , & De Vita, P. (2026). Multi‐trait and multi‐environment genomic prediction enhances yield components improvement in durum wheat. Frontiers in Plant Science, 17, 10.3389/fpls.2026.1759897 PMC12968193 41809631

[tpg270332-bib-0026] Saludares, R. A. , Atanda, S. A. , Piche, L. , Worral, H. , Dariva, F. , McPhee, K. , & Bandillo, N. (2024). Multi‐trait multi‐environment genomic prediction of preliminary yield trial in pulse crop. The Plant Genome, 17(3), e20496. 10.1002/tpg2.20496 PMC12807365 39099220

[tpg270332-bib-0027] Sparks, A. H. (2018). nasapower: A NASA POWER global meteorology, surface solar energy and climatology data client for R. Journal of Open Source Software, 3(30), 1035. 10.21105/joss.01035

[tpg270332-bib-0028] Tiezzi, F. , de Los Campos, G. , Parker Gaddis, K. L. , & Maltecca, C. (2017). Genotype by environment (climate) interaction improves genomic prediction for production traits in US Holstein cattle. Journal of Dairy Science, 100(3), 2042–2056. 10.3168/jds.2016-11543 28109596

[tpg270332-bib-0029] USDA . (2025). North Carolina agricultural statistics, 2025 (Publication No. 225). USDA. https://www.nass.usda.gov/Statistics_by_State/North_Carolina/Publications/Annual_Statistical_Bulletin/AgStat/NCAgStatBook.pdf

[tpg270332-bib-0030] Ward, B. P. , Merrill, K. , Bulli, P. , Pumphrey, M. , Mason, R. E. , Mergoum, M. , Johnson, J. , Sapkota, S. , Lopez, B. , Marshall, D. , & Brown‐Guedira, G. (2021). Analysis of the primary sources of quantitative adult plant resistance to stripe rust in U.S. soft red winter wheat germplasm. The Plant Genome, 14(1), e20082. 10.1002/tpg2.20082 PMC12807305 33595199

[tpg270332-bib-0031] Winn, Z. J. , Lyerly, J. H. , Brown‐Guedira, G. , Murphy, J. P. , & Mason, R. E. (2023). Utilization of a publicly available diversity panel in genomic prediction of Fusarium head blight resistance traits in wheat. The Plant Genome, 16(3), e20353. 10.1002/tpg2.20353 PMC12807144 37194437

[tpg270332-bib-0032] Zhu, M. , Zheng, Z. , Liu, W. , Han, Y. , Mou, W. , Yin, T. , Dai, X. , Wu, H. , Yang, Y. , Zan, Y. , & Liu, J. (2026). MMGS: A novel genomic prediction framework to integrate genotype, environment and their interactions for multi‐environment breeding trials. Horticulture Research, 13(5), uhag035. 10.1093/hr/uhag035 PMC13150855 42111488

