# Supplementary material for: Reaction norm modeling of high‐dimensional genomic and environmental data improves prediction accuracy in winter wheat
Source: Plant Genome. 2026 Oct 27;19(4):e70332. doi: 10.1002/tpg2.70332 (PMC13714343; doi:10.1002/tpg2.70332)
Supplement: Supplementary file 1 [file TPG2-19-e70332-s001.docx]

***Supplemental material***

**Reaction Norm Modeling of High-Dimensional Genomic and Environmental Data Improves Prediction Accuracy in Winter Wheat**

Shailesh Raj Acharya^1^, Julian Garcia Abadillo Velasco^3^, Jeanette Lyerly^1^, Gina Brown-Guedira^2^, Diego Jarquin^3^, Nonoy Bandillo^1, *^

^1^ Department of Crop and Soil Sciences, North Carolina State University, Raleigh, North Carolina, USA

^2^ USDA-ARS SEA, Plant Science Research, Raleigh, North Carolina, USA

^3^ Department of Agronomy, University of Florida, Gainesville, Florida, USA

*^*^* Corresponding author: nbbandil@ncsu.edu

Pages: 11

Supplemental Tables: 4

Supplemental Figures: 6

**Supplemental tables**

**Supplemental table S1.** Latitude and longitude of coordinates of four field locations involved in this study.

| **Location** | **Latitude (ºN)** | **Longitude (ºW)** |
| --- | --- | --- |
| Clayton, NC | 35.67 | 78.49 |
| Kinston, NC | 35.29 | 77.57 |
| Salisbury, NC | 35.69 | 80.62 |
| Plymouth, NC | 35.87 | 76.66 |

**Supplemental table S2.** Broad sense heritability (H^2^) estimation across all environments for yield, test-weight, heading date and plant height. Heritability estimates are only reported where the available data supported reliable estimation. Entries left blank denote cases in which H^2^ could not be obtained due to insufficient data, limited replication or unsuccessful model fitting.

| **Environment** | **Yield** | **TestWt** | **HeadDate** | **PlantHt** |
| --- | --- | --- | --- | --- |
| CLAYTON2017 | 0.70 | 0.87 | 0.95 | 0.33 |
| CLAYTON2018 | 0.62 | 0.77 |  | 0.59 |
| CLAYTON2019 | 0.33 | 0.81 |  |  |
| CLAYTON2020 |  | 0.08 |  |  |
| CLAYTON2021 | 0.83 | 0.95 |  |  |
| CLAYTON2022 | 0.61 | 0.96 |  |  |
| KINSTON2017 | 0.60 | 0.21 | 0.86 |  |
| KINSTON2018 | 0.66 | 0.32 | 0.83 |  |
| KINSTON2019 | 0.47 | 0.48 | 0.92 | 0.81 |
| KINSTON2020 | 0.70 | 0.83 | 0.88 |  |
| KINSTON2021 |  | 0.33 | 0.87 |  |
| KINSTON2022 | 0.61 | 0.82 | 0.89 |  |
| KINSTON2023 | 0.70 | 0.91 | 0.35 |  |
| PLYMOUTH2020 | 0.64 | 0.84 |  |  |
| PLYMOUTH2021 | 0.60 | 0.43 |  |  |
| PLYMOUTH2022 | 0.86 | 0.67 |  |  |
| RALEIGH2017 | 0.87 | 0.90 |  | 0.75 |
| RALEIGH2019 | 0.53 | 0.91 | 0.87 | 0.78 |
| RALEIGH2020 | 0.73 | 0.92 | 0.96 |  |
| SALISBURY2017 | 0.82 | 0.94 |  |  |
| SALISBURY2019 | 0.80 | 0.78 |  | 0.89 |
| SALISBURY2020 | 0.63 | 0.89 |  | 0.88 |
| SALISBURY2021 | 0.56 | 0.85 |  |  |

**Supplemental table S3.** Variance partitioning across all five models for four traits. The abbreviation varE, varL, varG, varGE, varGO and varR represent variance for environment, line, genomic, genotype-by-environment interaction, genotype-by-weather covariate interaction and residuals respectively.

|  |  | **varE** | **varL** | **varG** | **varGE** | **varGO** | **varR** |
| --- | --- | --- | --- | --- | --- | --- | --- |
| **Head Date** | M1(E+L) | 78.7 | 6.8 |  |  |  | 14.5 |
|  | M2(E+L+G) | 76.8 | 1.9 | 9.6 |  |  | 11.8 |
|  | M3(E+L+G+GE) | 77.1 | 1.7 | 6.3 | 9.1 |  | 5.7 |
|  | M4(E+L+G+GO) | 77.3 | 1.5 | 7.3 |  | 6.9 | 7.0 |
|  | M5(E+L+G+GE+GO) | 78.0 | 1.4 | 6.2 | 6.1 | 2.9 | 5.4 |
|  |  |  |  |  |  |  |  |
|  |  |  |  |  |  |  |  |
| **PlantHt** | M1(E+L) | 46.1 | 39.2 |  |  |  | 14.8 |
|  | M2(E+L+G) | 47.6 | 18.2 | 20.5 |  |  | 13.6 |
|  | M3(E+L+G+GE) | 40.7 | 15.2 | 26.9 | 8.9 |  | 8.3 |
|  | M4(E+L+G+GO) | 43.7 | 12.4 | 25.1 |  | 6.3 | 12.4 |
|  | M5(E+L+G+GE+GO) | 47.4 | 12.7 | 21.7 | 7.2 | 4.6 | 6.3 |
|  |  |  |  |  |  |  |  |
|  |  |  |  |  |  |  |  |
| **TestWt** | M1(E+L) | 55.4 | 22.2 |  |  |  | 22.3 |
|  | M2(E+L+G) | 51.1 | 7.8 | 19.6 |  |  | 21.5 |
|  | M3(E+L+G+GE) | 51.2 | 9.1 | 16.5 | 10.6 |  | 12.5 |
|  | M4(E+L+G+GO) | 48.7 | 6.7 | 17.3 |  | 10.9 | 16.4 |
|  | M5(E+L+G+GE+GO) | 49.2 | 8.7 | 16.3 | 7.0 | 5.9 | 12.9 |
|  |  |  |  |  |  |  |  |
|  |  |  |  |  |  |  |  |
| **Yield** | M1(E+L) | 55.9 | 14.3 |  |  |  | 29.8 |
|  | M2(E+L+G) | 54.8 | 3.4 | 13.2 |  |  | 28.6 |
|  | M3(E+L+G+GE) | 54.1 | 3.6 | 11.2 | 14.6 |  | 16.5 |
|  | M4(E+L+G+GO) | 52.0 | 2.7 | 10.1 |  | 13.7 | 21.4 |
|  | M5(E+L+G+GE+GO) | 53.5 | 3.0 | 10.5 | 10.6 | 5.9 | 16.4 |

**Supplemental table S4.** Prediction accuracies across all five models under each cross-validation schemes estimated for all four traits. The abbreviations E, L, G, GE and GO represent environment, line, genomic, genotype-by-environment interaction and genotype-by-weather covariate interaction respectively.

|  | **MODELS** | **CV00** | **CV0** | **CV1** | **CV2** |
| --- | --- | --- | --- | --- | --- |
|  |  |  |  |  |  |
|  | M1 (E + L) | 0.003 | 0.268 | -0.114 | 0.360 |
|  | M2 (E + L+ G) | 0.204 | 0.310 | 0.333 | 0.440 |
| **YIELD** | M3 (E + L + G + GE) | 0.182 | 0.277 | 0.411 | 0.500 |
|  | M4(E + L + G + GO) | 0.184 | 0.290 | 0.390 | 0.491 |
|  | M5(E +L+G+GE+GO) | 0.172 | 0.282 | 0.413 | 0.504 |
|  |  |  |  |  |  |
|  | M1 (E + L) | -0.032 | 0.430 | -0.263 | 0.433 |
|  | M2 (E + L+ G) | 0.102 | 0.463 | 0.137 | 0.504 |
| **PLANTHT** | M3 (E + L + G + GE) | 0.107 | 0.458 | 0.055 | 0.504 |
|  | M4(E + L + G + GO) | 0.037 | 0.422 | 0.143 | 0.490 |
|  | M5(E +L+G+GE+GO) | 0.140 | 0.402 | 0.134 | 0.504 |
|  |  |  |  |  |  |
|  | M1 (E + L) | 0.001 | 0.311 | -0.093 | 0.411 |
|  | M2 (E + L+ G) | 0.156 | 0.340 | 0.275 | 0.461 |
| **TESTWT** | M3 (E + L + G + GE) | 0.139 | 0.314 | 0.376 | 0.540 |
|  | M4(E + L + G + GO) | 0.157 | 0.347 | 0.355 | 0.530 |
|  | M5(E +L+G+GE+GO) | 0.150 | 0.337 | 0.378 | 0.554 |
|  |  |  |  |  |  |
|  | M1 (E + L) | 0.000 | 0.091 | -0.101 | 0.309 |
|  | M2 (E + L+ G) | 0.141 | 0.185 | 0.449 | 0.512 |
| **HEADDATE** | M3 (E + L + G + GE) | 0.095 | 0.186 | 0.493 | 0.555 |
|  | M4(E + L + G + GO) | 0.116 | 0.135 | 0.487 | 0.551 |
|  | M5(E +L+G+GE+GO) | 0.090 | 0.141 | 0.488 | 0.553 |

**Supplemental figures**

**Supplemental figure S1.** Total number of overlapping genotypes between environments for *‘TestWt’, HeadDate and Plant Height.* The matrix shows the number of lines in each environment (location-year combinations) on the diagonal. The upper off-diagonal shows common lines between environments, while the lower off-diagonal shows lines not shared between environments.

**Supplemental figure S2.** Schematics of four cross validation schemes to

evaluate prediction accuracy of five different models (M1 – M5).

**Supplemental figure S3.** Genome-wide distribution and density of filtered SNPs. The plot illustrates the chromosomal locations and local marker density of 40k SNPs mapped across the A, B and D sub genomes. Physical positions are indicated as megabases (Mb) along the horizontal axis, and color intensity corresponds to the local concertation of markers across the genome.

**Supplemental figure S4.** Genetic correlation between traits. Scatterplot matrix showing relationship among yield, test-weight, heading date and plant height. The diagonal panels represent the distribution of each variable as density curves. The lower triangular panel shows pairwise scatterplots and the upper panel represent the corresponding correlation coefficients between trait pairs.

**Supplemental figure S5.** PCA derived from NASA weather data. Color denotes the year associated with the environment. The first 2 PC explained 41.2% and 28.5% of the variance, respectively. 85% and 97% of the variance was explained with 3 and 5 PCs, respectively.

**Supplemental figure S6.** Mean Squared Error (MSE) achieved by the models under different cross-validation scenarios (columns) and predicting different traits (rows). In each grid, MSE is denoted in the y-axis, while models are denoted in the x-axis and color. The y-axis is scaled for each trait.
